# Comparison of Objectively Assessed Versus Patient-Reported Clarity of Last Rectal Effluent for the Prediction of Quality of Bowel Preparation for Colonoscopy: A Prospective, Case-Control Study

**DOI:** 10.7759/cureus.53828

**Published:** 2024-02-08

**Authors:** Ajay Patwa, Satish Kumar, Deepak Bhagchandani, Amit Kumar, Virendra Atam, Navneet Anil, Priya Mishra, Abhishek Singh, Archana Devi, Ajay K Pal

**Affiliations:** 1 Medicine, Gastroenterology and Hepatology Unit, King George’s Medical University, Lucknow, IND; 2 Medicine, King George’s Medical University, Lucknow, IND; 3 Internal Medicine, King George’s Medical University, Lucknow, IND; 4 Community Medicine & Public Health, King George’s Medical University, Lucknow, IND; 5 Surgery, King George’s Medical University, Lucknow, IND

**Keywords:** bowel preparation quality, photographic recording, cecal intubation time, procedure time, colonoscopy

## Abstract

Introduction: Colonoscopy is a crucial procedure for various clinical purposes, including screening for colorectal cancer. Adequate bowel preparation is essential for its success. Poor bowel preparation can lead to bad outcomes. An objective assessment of bowel preparation quality is typically only possible after the colonoscope is inserted. This study aimed to objectively correlate the clarity of last rectal effluent, directly collected in a transparent container, with the quality of bowel preparation, and compare it with patient-reported descriptions.

Methods: This prospective, single-centre, case-control study obtained ethical clearance and included patients aged >18 years undergoing colonoscopies. Cases included patients who collected the last rectal effluent and took photographs, while controls relied on verbal descriptions. Data collected included demographics, clinical information, bowel preparation quality, and lastly, stool clarity. A statistical analysis was performed to identify correlations and associations.

Results: Of the 70 included patients, 45 were male. The mean age was 35.8 ± 14.3 years. Cases had a higher mean age (37.8 ± 14.6). A higher number of cases had comorbidities (11, 68.8%). Photographic recording of the last rectal effluent was not associated with the adequacy of bowel preparation. Thin yellow fluid was the most common last-rectal effluent clarity (33, 47.1%). Thin, clear fluid was significantly associated with adequate bowel preparation.

Conclusion: Objective assessment of last rectal effluent clarity correlates with the quality of bowel preparation. This can improve the quality of bowel preparation for colonoscopies and potentially reduce the need for repeat procedures, contributing to better patient outcomes and cost savings in healthcare systems.

## Introduction

Colonoscopy is a time-consuming and expensive procedure. Ensuring adequate bowel preparation is a necessity for all colonoscopies. The quality of bowel preparation plays a key role in the technical as well as clinical success of the procedure. However, despite the best efforts, about 25% of patients have poor bowel preparation [1]. This may lead to abandonment, premature termination, missed clinical findings, and increased chances of repeat procedures [2]. Inadequate bowel preparation is also associated with increased procedure time and adverse events [3,4]. This leads to not only discomfort for the patient, endoscopy staff, and relatives but also an increased cost burden on the healthcare system. The worst part is that the actual assessment of the quality of bowel preparation can be done only after the colonoscope has been inserted inside. Although patient-described clear liquid stool and brown liquid last rectal effluent have been described as independent predictors for good and inadequate bowel preparation, respectively [5]. However, the patient’s description of rectal effluent may not be reliable, despite best efforts to explain [6]. In a study, photographs of the last rectal effluent taken from a commode-type toilet were correlated with the quality of bowel preparation [7]. But flush water used in toilets may dilute the last rectal effluent, hampering an accurate assessment of clarity. Besides, many parts of the world do not use commode-type toilets. So, we planned a case-control study primarily aimed at correlating the objectively assigned clarity code of the stool effluent, directly collected from the anal output in a transparent plastic container, with the quality of bowel preparation. We also aimed to compare it with the clarity reported verbally so that, if needed, a patient may be asked to take more bowel preparatory agents to improve preparation quality. In our pilot observational study, we showed the feasibility of this [8]. The secondary aims were to correlate total procedure time with the clarity code of the stool effluent, the quality of bowel preparation, and bowel findings in the colonoscopy.

## Materials and methods

Ethical clearance

Institutional ethics committee clearance was obtained (No.: 1357/Ethics/2021). Patients were not subjected to any additional tests, risks, procedures, or endoscopy time. However, informed consent was obtained from each patient for this study for the collection of the last rectal effluent.

Study setting

The study was performed in the endoscopy laboratory of the Department of Medicine at King George's Medical University Hospital.

Study design

**Figure 1:**
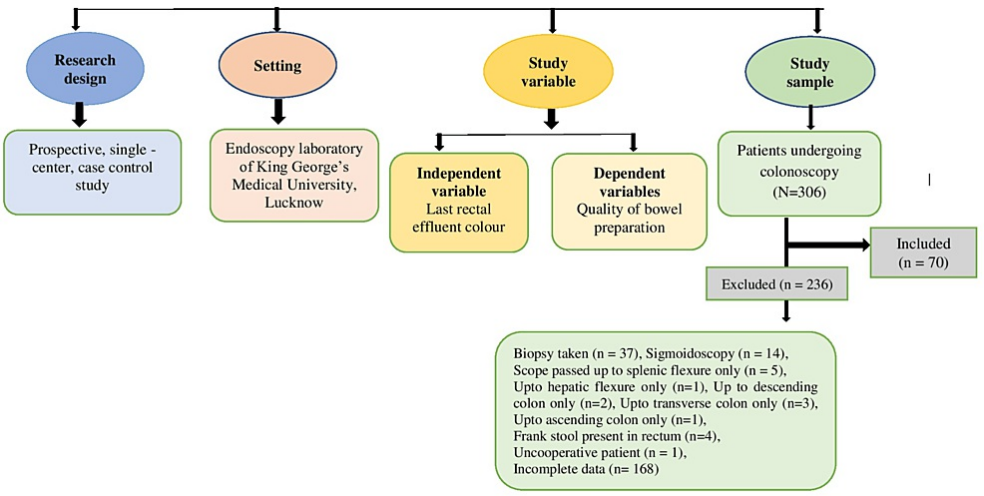
Schematic presentation of research design. Research design, setting, study variable (independent and dependent variables), and study sample (excluded 236 and included 70 patients).

This was a prospective, single-centre, cross-sectional, case-control study to objectively examine the rectal effluent clarity in patients undergoing colonoscopies to assess the quality of bowel preparation. One experienced endoscopist, assisted by one trainee endoscopist, performed the colonoscopies (Figure 1).

Study population

Inclusion Criteria

All patients aged >18 years, giving positive consent for colonoscopies and rectal effluent collection, were included in the study. Patients were provided written and verbal instructions from endoscopy nurses at the time of booking for colonoscopy regarding the bowel preparation regimen and fasting times based on the time of their procedure.

Cases

Patients who reported compliance with bowel preparation (those consuming >75% of the bowel preparatory agent) and taking photographs of the last rectal effluent were included as cases.

Controls

Patients who reported compliance with bowel preparation (those consuming >75% of the bowel preparatory agent) and not taking photographs of the last rectal effluent, only verbally reporting clarity of the last rectal effluent, were included as controls.

Exclusion Criteria

Patients with unstable vitals, pregnancy, not being ready to collect rectal effluent in a plastic container, colonoscopies not being examined up to the cecum, and those undergoing any therapeutic or diagnostic procedures, e.g., biopsy and polypectomy, were excluded.

Sample Size

The sample size was calculated using G*Power software (version 3.1). To detect the least clinically significant difference of 35% with a one-sided confidence level of 95% and a desired power of (80%), the required total sample size was 63. Further, the required sample size for controls was 21 and for cases was 42, with an allocation ratio of 2.

Data collection

Demographic data, including age, gender, and contact details, were recorded. Relevant clinical data, including indications of colonoscopy, comorbid illnesses, e.g., diabetes mellitus (DM), hypertension (HTN), tuberculosis (TB), coronary artery disease (CAD), chronic obstructive pulmonary disease (COPD), and cerebrovascular accidents (CVA), details of any surgical procedure, and a detailed history of any drug intake, were noted. Fasting time, last food consumed, amount of bowel preparatory agent consumed and its runway time, quality of bowel preparation, and objectively assigned clarity of the last rectal effluent were noted. Total procedure time, including cecal intubation time, cecal extubation time, and findings in colonoscopy, were also recorded.

Statistical analysis

Continuous and categorical variables related to the patient’s demographics, clinical data related to bowel preparation, and colonoscopic findings were presented as mean + SD and percentages, respectively. Two-sample t-tests, chi-square tests, and Fisher’s exact tests are used to test the independence of attributes and analyse the effect of these parameters on the quality of bowel preparation. A P-value of <0.05 was considered statistically significant.

Standard operating procedures and definitions

Last Rectal Effluent

It refers to the rectal effluent of the patient collected in a transparent plastic container before starting the colonoscopy.

Bowel Preparation

Written and verbal instructions were given to all patients. Patients were asked to take liquid food 24 hours prior to the colonoscopy. The types of bowel preparation agents used in the study were polyethylene glycol-based liquids. To calculate runway time, the last dose of the bowel preparation agent and the starting time of the colonoscopy were recorded as described in another study [9].

Assessment of the Quality of Bowel Preparation

**Figure 2:**
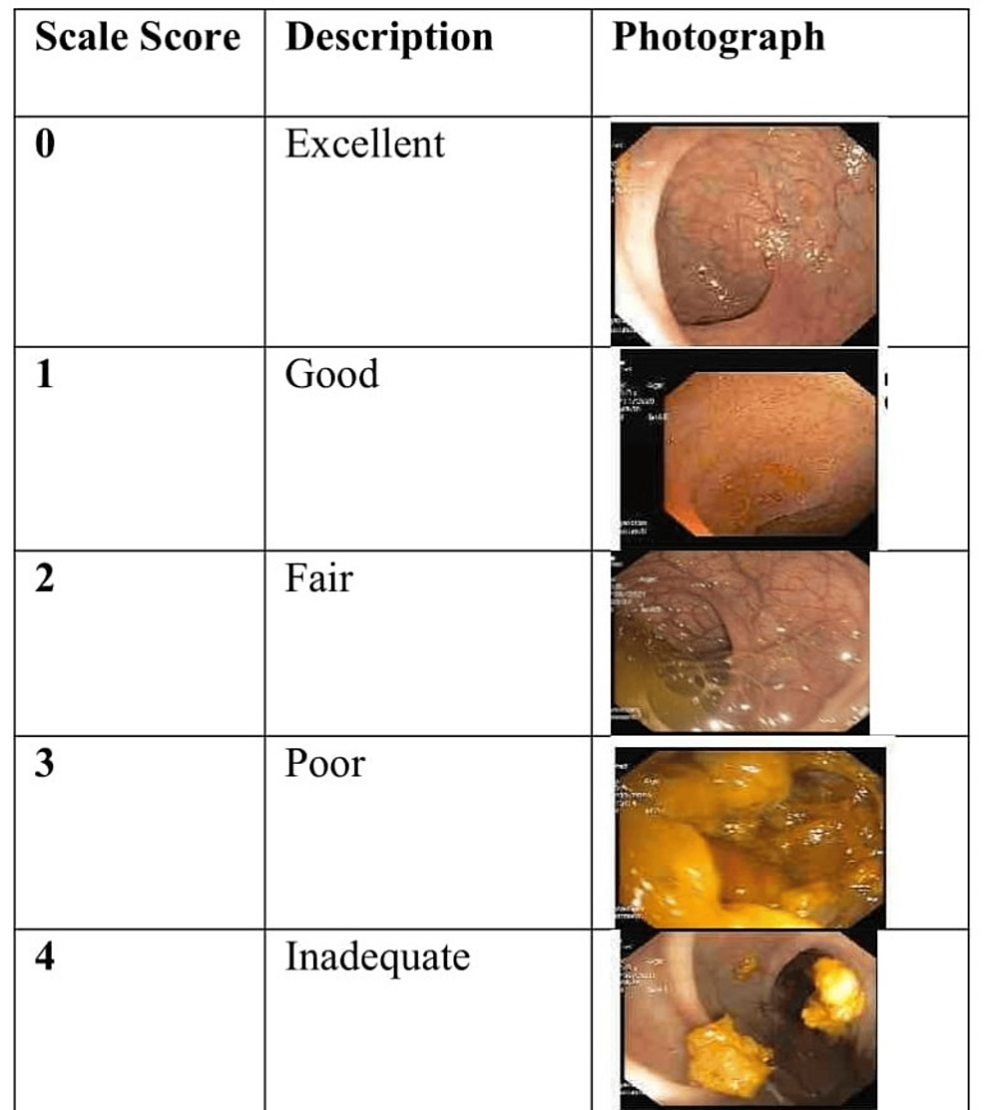
The Ottawa Bowel Preparation Scale. The Ottawa Bowel Preparation Scale was labelled as follows: 0 = excellent (mucosa clearly visible, almost no stool/fluid residual); 1 = good (some stool/fluid residue but mucosa visible even without suctioning); 2 = fair (some turbid stool/fluid but mucosa visible with some suctioning); 3 = poor (stool obscuring view of mucosa but reasonable view obtained with suctioning and washing); and 4 = inadequate (solid stool obscuring mucosa and not cleared with suctioning and washing).

The Ottawa Bowel Preparation Scale [10], a validated bowel preparation scale, was used to assess the adequacy of bowel preparation. Before using this scale, we performed a calibration exercise on the 11 patients selected as a pilot study [8]. The instrument requires endoscopists to rate the adequacy of three segments (right colon, mid colon, and rectosigmoid colon) on a scale as follows: 0 = excellent (mucosa clearly visible, almost no stool/fluid residual); 1 = good (some stool/fluid residue but mucosa visible even without suctioning); 2 = fair (some turbid stool/fluid but mucosa visible with some suctioning); 3 = poor (stool obscuring view of mucosa but reasonable view obtained with suctioning and washing); and 4 = inadequate (solid stool obscuring mucosa and not cleared with suctioning and washing) (Figure 2).

Since the right colon, mid colon, and rectosigmoid were graded separately according to the Ottawa scale as excellent, good, fair, poor, and inadequate, to categorise overall preparation as adequate or inadequate, the weight was given to the middle (if all three had different grades) or worst (if two regions had the same grade and the third different grade among all the three regions). For example, if the right colon is poor, the mid colon is fair, and the left colon is good, then overall preparation will be fair. The second example is that if the right colon is poor and the mid and left colons are fair, the overall preparation will be poor.

Collection of the Last Stool Effluent, Photographic Recording, and Assignment of Clarity

Patients were asked to collect at least 10 ml of the last rectal effluent in a 50-ml transparent plastic container and bring it to the endoscopy team before starting the colonoscopy. Photographs were taken with a phone camera with at least a 10-megapixel resolution capacity. The clarity of the rectal effluent was examined and labelled by a common consensus among the team comprising the above two endoscopists, two trained nursing staff, and three paramedical staff.

**Figure 3:**
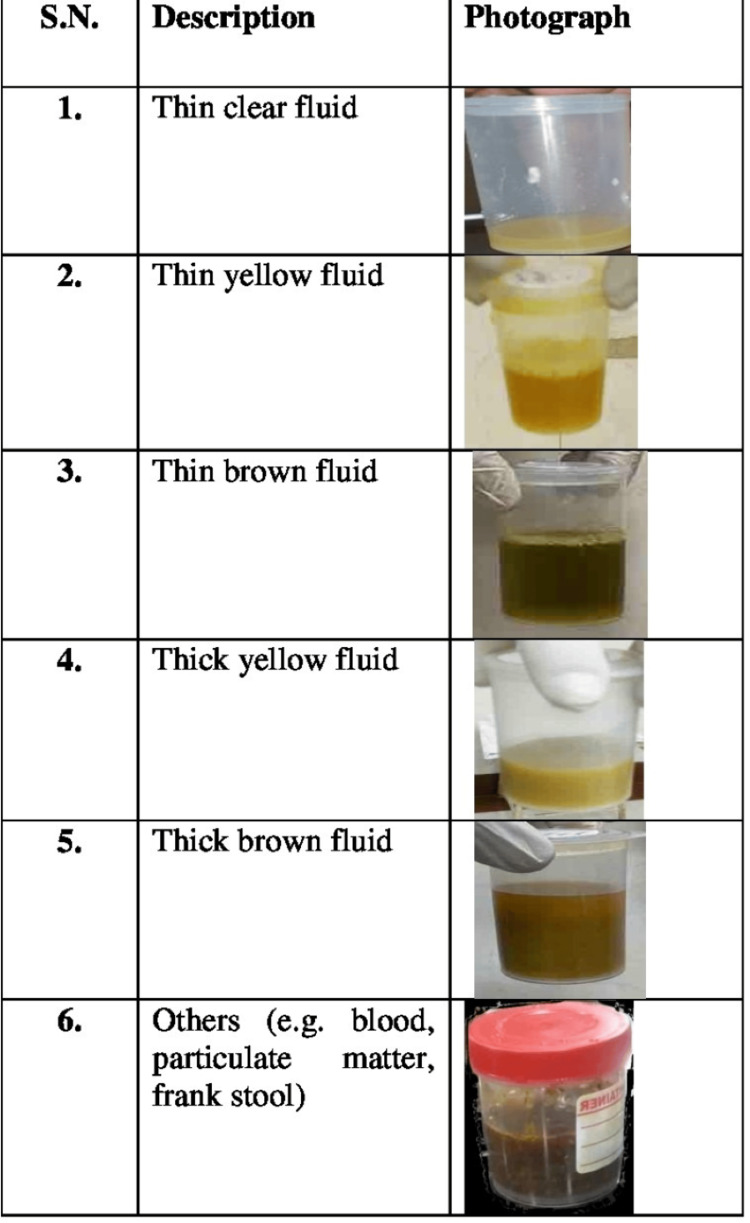
Last rectal effluent’s clarity scale. The clarity of the last rectal effluent was labelled as follows in order of increasing density and decreasing clarity: 1 = thin clear fluid; 2 = thin yellow fluid; 3 = thin brown fluid; 4 = thick yellow fluid; 5 = thick brown fluid; and 6 = others (e.g., blood and particulate matter).

The clarity of the rectal effluents was labelled as follows in order of increasing density and decreasing clarity: 1 = thin clear fluid; 2 = thin yellow fluid; 3 = thin brown fluid; 4 = thick yellow fluid; 5 = thick brown fluid; and 6 = others (e.g., blood and particulate matter). Reference points for the clarity of the rectal effluent were set from those of the 11 patients taken for the pilot study [8] after slight modification (Figure 3). In the current scale, those with solid stool were removed, and others (e.g., blood and particulate matter) were added. Those in others were analysed and discussed separately. Those not taking photographs (controls) were asked verbally about the clarity of the last rectal effluent and were assigned the same labels as above.

## Results

From January 2019 to April 2022, 306 colonoscopies were performed in our endoscopy unit. Among them, 70 were included in the study. Others were excluded due to various reasons, as described in Figure 1.

Baseline patient characteristics

The study was predominantly composed of males, accounting for 45 (64.3%). A majority of the patients (54, 77.1%) did not have any comorbidities, and 63 (90%) had no clinical addiction. The most common indications for the procedure fell under the miscellaneous category, with 22 (31.4%) cases encompassing conditions such as duodenal polyp (1), abdominal tuberculosis (5), inflammatory bowel diseases (7), liver metastasis (2), severe anaemia (4), generalised body weakness (2), lower abdominal mass (1), anal fissure (1), inflammatory bowel disease-related stricture (1), and chronic diarrhoea (1). This was followed by constipation in 17 (24.3%) cases and abdominal pain in 16 (16.9%) cases. The majority of patients exhibited normal colonoscopy findings, totalling 53 (75.7%), while nine (12.9%) patients presented with internal haemorrhoids. In terms of bowel preparation, 42 (60%) patients had inadequate preparation, whereas 28 (40%) had adequate preparation. Regarding the clarity of the last rectal effluent, 33 (47.1%) patients reported thin yellow fluid, 17 (24.3%) reported thin clear fluid, eight (11.4%) reported thin brown fluid, six (8.6%) reported thick brown fluid, and one (1.4%) reported frank stool (Table 1).

**Table 1:** Comparison between controls and cases by demographic, clinical findings, colonoscopy findings, bowel preparation scale, and stool clarity findings. Two sample t-test, chi-square test, and Fisher’s exact tests are used to test the independence of attributes. P-value was considered significant at <0.05.

Variables	Photograph of stool not taken: controls (N = 24, 34.2%)	Photograph of stool taken: cases (N = 46, 65.8%)	p-value
Demographic variables			
Age (mean ± SD), years	32.0 ± 13.3	37.8 ± 14.6	0.103
Gender			
Male	15 (33.3%)	30 (66.7%)	1.000
Female	9 (36.0%)	16 (64.0%)	
Clinical findings			
Any comorbidity			
No	19 (35.2%)	35 (64.8%)	1.000
Yes	5 (31.2%)	11 (68.8%)	
Clinical addiction			
No	23 (36.5%)	40 (63.5%)	0.409
Yes	1 (4.3%)	6 (85.7%)	
Indication			
None	1 (50.0%)	1 (50.0%)	0.970
Abdominal pain	5 (31.2%)	11 (68.8%)	
Altered bowel habit	2 (33.3%)	4 (66.7%)	
Constipation	5 (29.4%)	12 (70.6%)	
GI bleed	2 (28.6%)	5 (71.4%)	
Miscellaneous	9 (40.9%)	13 (59.1%)	
Colonoscopy findings			
Normal	16 (30.2%)	37 (69.8%)	0.438
Internal haemorrhoids	4 (44.4%)	5 (55.6%)	
Miscellaneous	4 (50.0%)	4 (50.0%)	
Bowel preparation			
Adequate	9 (37.5%)	19 (45.2%)	0.425
Inadequate	15 (62.5%)	27 (64.2%)	
Stool clarity			
Thick brown fluid	1 (16.7%)	5 (53.3%)	0.497
Thick yellow fluid	0 (0%)	5 (100.0%)	
Thin brown fluid	3 (37.5%)	5 (62.5%)	
Thin yellow fluid	13 (39.4%)	20 (60.6%)	
Thin clear fluid	7 (41.2%)	10 (58.8%)	
Others (Frank stool)	0 (0%)	1 (100.0%)	
Amount of bowel preparatory agent consumed (bottles) (mean ± SD, median, range)	(3.15 ± 1.71), 2, 2.85	(1.98 ± 1.39), 2, 0.5	0.003, t-test
Runway time (minutes) (mean ± SD, median, range)	(401.04 ± 19.68), 407.5, 450	(451.26 ± 21.01), 427.5, 760	0.000, t-test
Cecal intubation time (minutes) (mean ± SD, median, range)	(7.08 ± 2.60), 7, 11	(7.86 ± 2.77), 7, 25	0.258, t-test
Cecal extubation time (minutes) (mean ± SD, median, range)	(4 ± 4.8), 3, 8	(4.63 ± 2.12), 4, 16	0.448, t-test
Total procedure time (minutes) (mean ± SD, median, range)	(11.82 ± 3.39), 11, 23	(12.04 ± 3.43), 11, 26	0.799, t-test

Comparison of cases and controls

Cases had a higher mean ± SD of age as compared to controls (37.8 ± 14.6 versus 32.0 ± 13.3 years). In both genders, a higher number of patients took the last rectal effluent photograph (cases) (30, 66.7% vs. 16, 64.0%; male vs. female) as compared to those who did not take photographs (controls) (9, 36.0% vs. 15, 33.3%). Cases outnumbered controls for the presence of comorbidity (11, 68.8% vs. 5, 31.2%). Most of the cases and control had miscellaneous indications (13, 59.1% and 9, 40.9%), followed by constipation (12, 70.6% and 5, 29.4%) and abdominal pain (11, 68.8% and 5, 31.2%) for undergoing colonoscopy. Cases consumed significantly lower amounts of bowel preparatory agents. The runway time was significantly higher in the cases. Most of the cases and controls had normal colonoscopy findings (37, 69.8% vs. 16, 30.2%), followed by internal haemorrhoids (5, 55.6% and 4, 44.4%) and miscellaneous (4, 50.0% and 4, 50.0%). Cases were outnumbered in both adequate and inadequate bowel preparation groups. This finding was statistically not significant (p = 0.425), which is more than p-value <0.05. Frank stool was present in only one of the cases, so it was excluded from further data analysis. Most of the cases and controls had thin yellow fluid (20, 60.6% and 13, 39.4%) and thin clear fluid (10, 58.8% and 7, 41.2%) as their last rectal effluent. Cecal intubation, extubation, and total procedure time were not significantly different in cases and controls (Table 2).

**Table 2:** Distribution of patients by demographic, clinical findings, colonoscopy findings, bowel preparation scale, and stool clarity findings. * Any comorbidity includes diabetes, hypertension, tuberculosis, heart attack, stroke, paralysis, and chronic obstructive pulmonary disease. ** BMI (kg/m2) findings were based on data from 64 patients. P-value was considered significant at <0.05.

Variables	N = 70	% (CI)
Demographic variables		
Age (mean ± SD), years	35.8 ± 14.3	
Sex		
Male	45	64.3% (51.9-75.1%)
Female	25	35.7% (24.9-48.1%)
Clinical findings		
Any comorbidity*		
No	54	77.1% (65.3-86.0%)
Yes	16	22.9% (14.0-34.7%)
Clinical addiction		
No	63	90% (79.9-95.6%)
Yes	7	10% (4.5-20.1%)
Indications		
None	2	2.9% (0.5-10.9%)
Abdominal pain	16	22.9% (14.0-34.7%)
Altered bowel habits	6	8.6% (3.5-18.4%)
Constipation	17	24.3% (15.2-36.3%)
GI bleed	7	10% (4.5-20.1%)
Miscellaneous	22	31.4% (21.2-43.8%)
Body mass index (BMI)**	21.1±3.8	
Colonoscopy findings		
Normal	53	75.7% (63.7-84.8%)
Internal haemorrhoids	9	12.9% (6.4-23.5%)
Miscellaneous	8	11.4% (5.4-21.8%)
Bowel preparation		
Adequate	28	40% (30-60%)
Inadequate	42	60% (25-80%)
Last stool clarity		
Frank stool	1	1.4% (0.1-8.8%)
Thick brown fluid	6	8.6% (3.5-18.4%)
Thick yellow fluid	5	7.1% (2.7-16.6%)
Thin brown fluid	8	11.4% (5.4-21.8%)
Thin yellow fluid	33	47.1% (35.2-59.4%)
Thin clear fluid	17	24.3% (15.2-36.3%)
Amount of bowel preparatory agent consumed (mottles) (mean ± SD, median, range)	(11.97 ± 3.43), 2, 0.5	
Runway time (minutes) (mean ± SD, median, range)	(435.05 ± 6.10), 440, 860	
Cecal intubation time (minutes) (mean ± SD, median, range)	(7.44 ± 2.70), 7, 23	
Cecal extubation time (minutes) (mean ± SD, median, range)	(4.84 ± 2.06), 4, 12	
Total procedure time (minutes) (mean ± SD, median, range)	(11.97 ± 1.39), 11, 26	

Comparison between patients with adequate and inadequate bowel preparation

The adequate bowel preparation group had significantly higher age as compared to the inadequate bowel preparation group (39.67 ± 6.18 vs. 33.33 ± 5.70, p = 0.000) and had a significantly higher frequency of male gender patients (p = 0.042). The adequate bowel preparation group had a significantly higher frequency of thin clear fluid as the last rectal effluent, while the inadequate bowel preparation group had a higher frequency of thin yellow, thick brown, or thick yellow fluid (p = 0.003). The adequate bowel preparation group had a significantly longer runway time (p = 0.001). Cecal intubation time was significantly shorter in the adequate bowel preparation group (p = 0.006), which ultimately resulted in a shorter procedure time (Table 3).

**Table 3:** Comparison between adequate and inadequate bowel preparation as per demographic, clinical findings, colonoscopy findings, bowel preparation scale, and stool clarity findings. * Any comorbidity includes diabetes, hypertension, tuberculosis, heart attack, stroke, paralysis, and chronic obstructive pulmonary disease. P-value was considered significant at <0.05.

Variables	Adequate bowel preparation (N = 28, 40%)	Inadequate bowel preparation (N = 42, 60%)	p-value, test
Demographic variables			
Age (mean ± SD), years	39.67 ± 6.18	33.33 ± 5.70	0.000, t-test
Gender			
Male	18 (64.28%)	28 (66.66%)	0.042, chi-square test
Female	10 (35.71%)	14 (33.33%)	
Clinical findings			
Any comorbidity*			
No	26 (92.85%)	40 (95.23%)	1.000, Fisher’s exact test
Yes	2 (7.14%)	2 (4.76%)	
Clinical addiction			
No	25 (89.28%)	38 (90.47%)	1.000, Fisher’s exact test
Yes	3 (10.71%)	4 (9.52%)	
Indication			
None	12 (42.85%)	1 (2.38%)	0.000, chi-square test
Abdominal pain	7 (25%)	8 (19.04%)	
Altered bowel habit	0 (0%)	6 (14.28%)	
Constipation	7 (28%)	9 (21.42%)	
GI bleed	2 (7.15%)	4 (9.52%)	
Miscellaneous	0 (0%)	14 (33.33%)	
BMI	20.22 ± 4.41	18.54 ± 4.26	0.68, t-test
Colonoscopy findings			
Normal	16 (57.14%)	36 (85.71%)	0.026, chi-square test
Internal haemorrhoids	7 (25%)	4 (9.52%)	
Miscellaneous	5 (17.85%)	2 (4.76%)	
Stool clarity			0.003, chi-square test
Thick brown fluid	3 (10.71%)	3 (7.14%)	
Thick yellow fluid	0 (0%)	5 (11.90%)	
Thin brown fluid	1 (3.57%)	7 (16.66%)	
Thin yellow fluid	10 (35.71%)	22 (52.38%)	
Thin clear fluid	14 (50%)	5 (11.90%)	
Cases versus controls			
Cases	18 (64.28%)	29 (69.04%)	0.678, chi-square test
Controls	10 (35.71%)	13 (30.95%)	
Amount of bowel preparatory agent consumed (bottles) (mean ± SD, median, range)	(1.96 ± 1.37), 2, 0.5	(1.97 ± 1.38), 2, 0.5	0.976, t-test
Runway time (minutes) (mean ± SD, median, range)	(445.75 ± 20.73), 483, 418	(428.11 ± 20.44), 411, 760	0.001, t-test
Cecal intubation time (minutes) (mean ± SD, median, range)	(6.46 ± 2.49), 6.5, 10	(8.35 ± 2.85), 7.5, 25	0.006, t-test
Cecal extubation time (minutes) (mean ± SD, median, range)	(4.64 ± 2.11), 4, 16	(4.26 ± 2.03), 3, 12	0.453, t-test
Total procedure time (minutes) (mean ± SD, median, range)	(11.14 ± 3.27), 10, 20	(12.61 ± 3.50), 11, 24	0.082, t-test

Comorbidities

Various comorbidities and their percentages in the study population were as follows: DM (2, 2.86%), HTN (6, 8.57%), TB (6, 8.57%), and CAD (2, 2.86%). There was no significant difference between adequate and inadequate bowel preparation groups in relation to the presence of comorbidities.

## Discussion

Our study aimed to investigate the correlation between the objectively assessed clarity of rectal effluent and the quality of bowel preparation in patients undergoing colonoscopies. We also sought to compare these objective findings with the patient’s verbal descriptions of their rectal effluent. By doing so, we aimed to improve the accuracy of assessing bowel preparation quality before the colonoscopy procedure begins. In this discussion, we will interpret our findings, highlight the strengths and limitations of our study, and discuss their implications.

Among the key findings, we observed a significant correlation between the clarity of the last rectal effluent and the quality of bowel preparation (p = 0.003). These findings suggest that an objective assessment of the rectal effluent can serve as a reliable predictor of bowel preparation quality.

The quality of bowel preparation plays an important role in colonoscopy. Procedural adverse events during or after the colonoscopy are the result of poor bowel preparation and have been extensively investigated [11,12]. The results of our study are consistent with previous research that has demonstrated a significant correlation between the quality of bowel preparation and the success of colonoscopy procedures. For example, a study by Harewood et al. (2003) found that patients with poor bowel preparation were more likely to have incomplete colonoscopies and lower adenoma detection rates [13]. Similarly, a study by Froehlich et al. (2005) found that patients with poor bowel preparation were more likely to have longer procedure times and lower adenoma detection rates [4]. Our study builds on this previous research by demonstrating the feasibility and reliability of objectively assessing bowel preparation quality through the collection and analysis of the last rectal effluent. While previous studies have relied on subjective assessments of bowel preparation quality, our study provides a more objective and reliable measure of bowel preparation quality. Furthermore, our study highlights the limitations of relying solely on patient-reported descriptions of rectal effluent. This finding is consistent with previous research that has suggested that patient-reported descriptions may not be reliable indicators of the actual quality of bowel preparation [4]. Therefore, we recommend that clinicians consider incorporating objective measures of bowel preparation quality, such as the collection and analysis of the last rectal effluent, into their standard practice. Overall, our study provides valuable insights into the importance of bowel preparation for successful colonoscopy procedures. By objectively assessing bowel preparation quality through the collection and analysis of the last rectal effluent, clinicians can improve the technical and clinical success of colonoscopy procedures and provide better care for their patients. Our findings are consistent with previous research and suggest that objectively assessing bowel preparation quality should be a standard practice in colonoscopy procedures.

Strengths and limitations

Our study presents several strengths, such as its prospective design and observational nature, making the results directly applicable to clinical practice. However, it is important to acknowledge the limitations of our study, which primarily revolve around a small sample size and single-centre study without external validation. This may have impacted the statistical power and generalisability of our findings. Additionally, the inconvenience of collecting rectal effluent in a plastic container and bringing it to the endoscopy team could deter some patients. Although we instructed patients not to collect effluent mixed with toilet water, ensuring compliance with these instructions may have posed some challenges. Future studies could explore alternative methods for objective assessment of rectal effluents, such as having patients or their relatives take photographs, provided proper instructions are given to avoid contamination.

Clinical implications

Our findings have important clinical implications. Ensuring adequate bowel preparation is crucial for the success of colonoscopy procedures, as inadequate bowel preparation can lead to missed clinical findings, prolonged procedure times, and the need for repeat colonoscopies. The use of an objective tool to assess the quality of bowel preparation, such as the clarity of the rectal effluent, can aid endoscopists in making informed decisions during the procedure.

## Conclusions

In conclusion, our study demonstrated a significant correlation between the objectively assessed clarity of rectal effluent and the quality of bowel preparation. These findings emphasize the importance of reliable predictors for bowel preparation quality, which can enhance the efficiency and outcomes of colonoscopy procedures. While our study had limitations, including a small sample size, future research can build upon these findings and explore alternative methods for objective assessment of rectal effluent to further improve the quality of colonoscopies and patient care.
